# Protocatechuic Acid Extends Survival, Improves Motor Function, Diminishes Gliosis, and Sustains Neuromuscular Junctions in the hSOD1^G93A^ Mouse Model of Amyotrophic Lateral Sclerosis

**DOI:** 10.3390/nu12061824

**Published:** 2020-06-18

**Authors:** Lilia A. Koza, Aimee N. Winter, Jessica Holsopple, Angela N. Baybayon-Grandgeorge, Claudia Pena, Jeffrey R. Olson, Randall C. Mazzarino, David Patterson, Daniel A. Linseman

**Affiliations:** 1 Department of Biological Sciences, F. W. Olin Hall, Room 102, University of Denver, 2190 E. Iliff Ave, Denver, CO 80208, USA; lilia.koza@du.edu (L.A.K.); anwinter@usf.edu (A.N.W.); jessie.h511@gmail.com (J.H.); bg.angela1@gmail.com (A.N.B.-G.); claudia.pena@du.edu (C.P.); jeffrey.olson.du@gmail.com (J.R.O.); randall.mazzarino@du.edu (R.C.M.); david.patterson@du.edu (D.P.); 2 Knoebel Institute for Healthy Aging, Engineering Computer Science, Suite 579, University of Denver, 2155 E. Wesley Ave, Denver, CO 80208, USA; 3 Eleanor Roosevelt Institute, University of Denver, 2101 E. Wesley Ave, Denver, CO 80210, USA

**Keywords:** amyotrophic lateral sclerosis, anti-inflammatory, antioxidant, phenolic acid, neuroprotective, neurodegeneration

## Abstract

Amyotrophic lateral sclerosis (ALS) is a devastating disorder characterized by motor neuron apoptosis and subsequent skeletal muscle atrophy caused by oxidative and nitrosative stress, mitochondrial dysfunction, and neuroinflammation. Anthocyanins are polyphenolic compounds found in berries that possess neuroprotective and anti-inflammatory properties. Protocatechuic acid (PCA) is a phenolic acid metabolite of the parent anthocyanin, kuromanin, found in blackberries and bilberries. We explored the therapeutic effects of PCA in a transgenic mouse model of ALS that expresses mutant human Cu, Zn-superoxide dismutase 1 with a glycine to alanine substitution at position 93. These mice display skeletal muscle atrophy, hindlimb weakness, and weight loss. Disease onset occurs at approximately 90 days old and end stage is reached at approximately 120 days old. Daily treatment with PCA (100 mg/kg) by oral gavage beginning at disease onset significantly extended survival (121 days old in untreated vs. 133 days old in PCA-treated) and preserved skeletal muscle strength and endurance as assessed by grip strength testing and rotarod performance. Furthermore, PCA reduced astrogliosis and microgliosis in spinal cord, protected spinal motor neurons from apoptosis, and maintained neuromuscular junction integrity in transgenic mice. PCA lengthens survival, lessens the severity of pathological symptoms, and slows disease progression in this mouse model of ALS. Given its significant preclinical therapeutic effects, PCA should be further investigated as a treatment option for patients with ALS.

## 1. Introduction

Amyotrophic lateral sclerosis (ALS), also known as Lou Gehrig’s disease, is a devastating, progressive, and fatal neurodegenerative disease that affects motor neurons of the central nervous system. ALS patients exhibit a median survival of only 2–3 years following diagnosis, with death typically caused by respiratory failure [1]. ALS presents as either a sporadic or familial disease. Sporadic ALS cases account for approximately 90% of all patients and do not have an obvious genetic cause. Familial ALS accounts for the remaining 10% of patients and has been linked to mutations in genes such as Cu, Zn-superoxide dismutase 1 (SOD1), chromosome 9 open reading frame 72 (C9orf72), fused in sarcoma, and TAR DNA-binding protein 43 (TDP-43) [2]. Although ALS is classified as a rare disease, with a prevalence of 5 in 100,000 people living in the United States, the effects of the disease are calamitous for those who are afflicted [1]. ALS is characterized pathologically by the death of motor neurons, axonal retraction away from the neuromuscular junctions (NMJs), skeletal muscle atrophy, and ultimately, death.

The pathogenesis underlying both familial and sporadic forms of ALS has been extensively studied but is still not completely understood. Protein aggregation, disrupted axonal transport, perturbed RNA metabolism, excitotoxicity, neuroinflammation, mitochondrial dysfunction, and oxidative stress have all been identified as underlying mechanisms and contributing factors in ALS. In the context of motor neuron degeneration, neuroinflammation and oxidative stress appear to be major pathogenic mechanisms. Both astrocytes and microglia can adopt distinct anti- or pro-inflammatory phenotypes, depending on signals from the surrounding environment. These anti- or pro-inflammatory phenotypes are neuroprotective or neurotoxic to motor neurons, respectively. Astrocytes, although beneficial to neurons in their resting state, become reactive and contribute to motor neuron death in various models of ALS [3,4,5,6]. Reactive astrocytes have also been shown to impair the process of autophagy in motor neurons, resulting in increased protein aggregation and reduced motor neuron health in in vivo and in vitro models of ALS [7,8,9]. Microglia also contribute significantly to the neuroinflammation in ALS. Microglia have been shown to induce astrocyte reactivity by releasing pro-inflammatory cytokines, resulting in an inability of astrocytes to protect motor neurons [10,11]. Microglia also become pro-inflammatory and have been found to display an ALS-specific phenotype that contributes to rapid disease progression and increased motor neuron loss [12,13,14,15].

Oxidative stress is another mechanism that has been identified as a causative factor in ALS. Increased oxidative stress burden correlates positively with disease severity [16,17]. Superoxide and nitric oxide have been found to be elevated in ALS [18,19]. Oxidative stress markers such as glutathione peroxidase, malondialdehyde, glutathione status, and 8-oxodeoxyguanosine demonstrate significant alterations in ALS patients [20,21]. Furthermore, mitochondrial dysfunction and mutations in genes that affect mitochondrial processes have been linked to ALS [22,23,24,25,26]. Specifically, in the case of ALS caused by mutations in SOD1, the mutant SOD1 protein has been shown to aggregate within mitochondria, resulting in mitochondrial dysfunction and mitochondrial oxidative stress [24,27]. Given the above findings, a beneficial therapeutic approach for ALS may be to reduce both neuroinflammation and oxidative stress.

Currently, only two drugs, Riluzole and Edaravone, have been approved by the U.S. Food and Drug Administration (FDA) to treat ALS. Riluzole is administered orally, only has a modest effect on slowing disease progression, and shows an extension of life of only a few months [2]. Edaravone has been shown to delay disease progression by only 33% when compared to a placebo, is costly, and must be administered intravenously [28]. Unfortunately, both Riluzole and Edaravone have only modest effects on disease progression and survival. Furthermore, both drugs are expensive and display substantial side effects. Many other therapeutics have failed in the clinic or are still undergoing clinical trials. However, no new pharmacological therapies are immediately on the horizon for ALS patients.

Nutraceuticals, natural bioactive compounds found in foods, may be safe, easy to administer, and cost-effective therapeutic treatments for ALS. More specifically, anthocyanins, a type of flavonoid, may be beneficial to ALS patients due to their substantial antioxidant and anti-inflammatory properties [29]. Anthocyanins vary in color from red to blue and are responsible for the vibrant coloring of many fruits and vegetables [30]. We have previously demonstrated a therapeutic effect of an anthocyanin-enriched strawberry extract in a transgenic mouse model of ALS that expresses mutant human SOD1 with a glycine to alanine substitution at position 93 (hSOD1^G93A^) [31]. This strawberry extract is enriched in callistephin, an anthocyanin derived from pomegranates and strawberries [29,32]. We have also shown that callistephin suppresses apoptosis induced by mitochondrial oxidative stress and protects neurons from glutamate excitotoxicity in vitro [29,32]. In vivo, we found that hSOD1^G93A^ mice treated with strawberry anthocyanin extract beginning at 60 days old showed delayed disease onset, improved grip strength throughout the disease, and significantly extended survival when compared to untreated hSOD1^G93A^ littermate mice. Furthermore, treated mice displayed significantly decreased astrogliosis in the spinal cord and preserved NMJs in gastrocnemius muscle when compared to untreated littermate mice [31]. These findings indicate that anthocyanin compounds may have therapeutic potential in ALS. However, despite their potential benefits, parent anthocyanins suffer from poor bioavailability. In contrast, their phenolic acid metabolites typically display much higher bioavailability and most can readily cross the blood brain barrier [33].

Here, we examined the therapeutic potential of protocatechuic acid (PCA) in the hSOD1^G93A^ preclinical mouse model of ALS. PCA is a phenolic acid metabolite of kuromanin, the parent anthocyanin found in blackberries, bilberries, and black rice. We have previously shown that kuromanin protects neurons from oxidative stress induced by glutamate excitotoxicity, nitrosative stress induced by nitric oxide, and it also suppresses mitochondrial oxidative stress and the consequent apoptosis by preserving mitochondrial glutathione [29,32]. In a similar manner, PCA protects neurons against nitrosative and oxidative stress and reduces nitric oxide production in microglial cells treated with lipopolysaccharide, demonstrating both antioxidant and anti-inflammatory activities [34]. Based on our previous findings, we tested the therapeutic effects of PCA in the hSOD1^G93A^ mouse model of ALS.

## 2. Materials and Methods

### 2.1. The hSOD1^G93A^ Mouse Model of ALS

FVB/NJ mice harboring a human transgene coding for a mutated form of SOD1 with a glycine to alanine substitution at position 93 were obtained from The Jackson Laboratory (Bar Harbor, ME, USA). Mice were bred and maintained at the University of Denver animal facility under a standard 12 h light/dark cycle with food and water provided ad libitum. Genotyping to identify transgenic mice was carried out by a third-party company, Transnetyx Inc. (Cordova, TN, USA). All procedures were performed in accordance with two protocols approved by the institutional animal care and use committee at the University of Denver. The initial protocol (927091) was approved on 21 July 2016 and the second protocol (1454889) was approved on 12 July 2019.

### 2.2. Survival Data

For the survival study, mice were divided into four groups consisting of 15 mice each. The first group consisted of non-transgenic wild-type (WT) age- and sex-matched littermate controls. The other three groups consisted of age- and sex-matched transgenic hSOD1^G93A^ littermate mice, either untreated or treated with either 50 or 100 mg/kg PCA. PCA was dissolved in sterile deionized water and was administered once per day as a 0.25 mL dose by oral gavage 5 days/week. Oral gavage treatment of PCA began at disease onset (90 days of age) and continued until mice reached end stage, defined as the point at which a mouse no longer had the ability to right itself within 15 s after being placed on its side. PCA-treated and untreated hSOD1^G93A^ littermate mice were euthanized at end stage by an overdose of inhaled isoflurane (Vet One, Boise, ID, USA). The WT littermate control mouse was euthanized at end stage of whichever hSOD1^G93A^ littermate mouse lived the longest, regardless of treatment. 

### 2.3. Paw Grip Endurance and Rotarod Testing

Hind limb strength was assessed by paw grip endurance (PaGE) testing twice per week beginning at disease onset. Briefly, mice were placed on top of a standard wire cage lid which was suspended a few inches above the bench top. Mice were briefly allowed to acclimate before the cage lid was smoothly inverted to prompt the mouse to grip the wire with both its fore and hind limbs. A stopwatch was started as soon as the cage lid was inverted and the time was measured to determine how long the mouse could hold on before its hind legs released their grip from the cage lid, expressed as latency to fall. Care was taken not to jostle the lid during this time. The stopwatch was stopped at a maximum of 30 s. Mice were given five scored attempts and the highest and lowest scores were excluded from the final score. Final scores are reported as an average of the three remaining scored attempts ± standard error of the mean (SEM) for each time point. Time points correspond to the age of the animal at the time that testing was performed and are reported as a range of several days since multiple sets of animals having slightly different ages were tested concomitantly.

Motor function and endurance were assessed by accelerating rotarod testing once per week beginning at disease onset. Mice were placed on a rod, 30 mm in diameter, rotating at 4 rpm. Each animal was placed in one lane and subjected to three trials. The lane was cleaned before the next mouse was tested to prevent interference. Once the mice were acclimated to the initial speed of 4 rpm, the rod was accelerated from 4 to 40 rpm over the course of 5 min. The time was stopped when the mouse fell off the rotating rod, expressed as latency to fall. Mice were given three scored attempts reported as an average ± SEM for each time point. Time points correspond to the age of the animal at the time that testing was performed and are reported as a range of several days since multiple sets of animals having slightly different ages were tested concomitantly.

### 2.4. Analysis of Mice at End Stage of the Untreated hSOD1^G93A^ Littermate Mouse

For assessment of Nissl-stained motor neuron counts, glial fibrillary acidic protein (GFAP) and ionized calcium-binding adapter molecule 1 (Iba-1) staining, NMJ area, perimeter, and Sholl analysis, mice were divided into three groups consisting of approximately 10 mice per group. The first group consisted of non-transgenic WT age- and sex-matched littermate controls. The other two groups consisted of age- and sex-matched transgenic hSOD1^G93A^ littermate mice, either untreated or treated with 100 mg/kg PCA orally as described above. PCA treatment continued until the untreated hSOD1^G93A^ littermate mouse reached end stage. At that point, mice from all 3 groups were euthanized and spinal cord and gastrocnemius muscles were collected. 

### 2.5. Analysis of Mice at 105 Days of Age

We observed the greatest improvements in motor function as assessed by rotarod and PaGE testing between 97 and 114 days of age in PCA-treated hSOD1^G93A^ mice. Therefore, we analyzed the gastrocnemius muscle wet weight, vesicular acetylcholine transporter (VAChT) and alpha-bungarotoxin (BTx) co-stained gastrocnemius muscle, and 4-hydroxynonenal (4-HNE)-stained spinal cord ventral horn from a separate cohort of mice at 105 days of age. This cohort consisted of both WT and hSOD1^G93A^ mice with or without PCA treatment, with each treatment group containing approximately 10 mice. Mice receiving PCA were given a daily dose of 100 mg/kg beginning at 90 days of age and continuing until the mice reached 105 days of age. At 105 days of age, mice from all 3 groups were euthanized and gastrocnemius muscles and lumbar spinal cord were collected.

### 2.6. Tissue Preparation and Cryosectioning

Following euthanasia, the thoracolumbar portion of the spinal column, containing the lumbar spinal cord, and gastrocnemius muscle were removed. Each specimen was washed with 1X phosphate-buffered saline (PBS, pH 7.4), and placed in 4% paraformaldehyde at 4 °C overnight. Each specimen was then washed again and allowed to sit in 1X PBS for 20 min. Gastrocnemius muscle was placed in 30% sucrose in 1X PBS and allowed to sink for cryoprotection. The lumbar spinal cord was placed in 6% trichloroacetic acid (Sigma-Aldrich, St. Louis, MO, USA) in deionized water for 6 days for decalcification. Following decalcification, the lumbar spinal cord was placed in 30% sucrose until saturated. Both gastrocnemius muscle and spinal cord were frozen rapidly in optimal cutting temperature (OCT) compound with liquid nitrogen and stored at −80 °C until sectioning took place. Prior to sectioning, tissue was allowed to acclimate in the microtome cryostat for at least 20 min. For gastrocnemius muscle and spinal cords, sections of 30 μm in length were cut and every viable tissue section was collected onto the surface of Fisherbrand Superfrost Colorfrost Plus coated slides (Fisher Scientific, Pittsburgh, PA, USA). Slides were stored at −20 °C until subjected to immunohistochemistry.

### 2.7. Immunohistochemistry of Spinal Cord Sections

Prior to staining, slides were allowed to equilibrate at room temperature for at least 30 min. Tissue sections on the slides were then outlined with a hydrophobic pen (Liquid Blocker Super PAP Pen; Daido Sangyo Co., Tokyo, Japan) and washed twice with 1X PBS to remove any residual OCT. Tissue was then incubated at room temperature in blocking buffer, containing 5% (*w*/*v*) bovine serum albumin (BSA) and 1X PBS containing 0.2% triton-X 100 for 90 min. For astrocyte staining, primary antibody to GFAP (Abcam, Cambridge, MA, USA) was then prepared as a 1:500 dilution in 1X PBS containing 0.2% triton-X 100 and 2% BSA (*w*/*v*). For microglial staining, primary antibody to Iba-1 (Abcam, Cambridge, MA, USA) was prepared as a 1:167 dilution in PBS containing 0.2% triton-X 100 and 2% BSA (*w*/*v*). For 4-HNE staining, primary antibody to 4-HNE (Alpha Diagnostic Intl. Inc., San Antonio, TX, USA) was prepared as a 1:500 dilution in PBS containing 0.2% triton-X 100 and 2% BSA (*w*/*v*). Tissue was incubated in primary antibody overnight at 4 °C. Tissue was washed 3–4 times with 1X PBS to remove any unbound primary antibody. FITC-conjugated donkey anti-rabbit antibody (Jackson Immunoresearch Laboratories, West Grove, PA, USA) or Alexa Fluor 488-conjugated donkey anti-goat antibody (Jackson Immunoresearch Laboratories, West Grove, PA, USA) were then prepared at 1:500 dilutions (*v*/*v*) in 1X PBS containing 0.2% triton-X100 and 2% BSA (*w*/*v*) and Hoechst nuclear stain (Sigma-Aldrich, St. Louis, MO, USA) at a 1:500 dilution to detect GFAP and Iba-1, and to label nuclei, respectively. For 4-HNE staining, FITC-conjugated donkey anti-rabbit antibody (Jackson Immunoresearch Laboratories, West Grove, PA, USA) was prepared at 1:500 dilution (*v*/*v*) in 1X PBS containing 0.2% triton-X100 and 2% BSA (*w*/*v*) and Hoechst nuclear stain (Sigma-Aldrich, St. Louis, MO, USA) at a 1:500 dilution to detect 4-HNE and to label nuclei, respectively. Sections were incubated with secondary antibodies at room temperature for 90 min, then washed 3–4 times with 1X PBS. ProLong Gold anti-fade reagent (Thermo Fisher Scientific, Eugene, OR, USA) was used as mounting medium and slides were sealed with coverslips. Stained slides were stored in the dark at −20 °C until imaging took place. Twelve sections of spinal cord were stained for Iba-1, GFAP, and 4-HNE per mouse.

### 2.8. Imaging and Quantification of Spinal Cord Sections Stained for Iba-1 and GFAP at End Stage of the Untreated hSOD1^G93A^ Littermate Mouse

Tissue was imaged using a Zeiss Axio Observer epifluorescence microscope to capture a single image of each ventral horn on the 20× objective. Imaging was performed by blinded researchers. For Iba-1 and GFAP, images were captured on the Alexa Fluor 488 channel and the exposure time was set appropriately for the untreated hSOD1^G93A^ littermate and kept constant when imaging the PCA-treated hSOD1^G93A^ and WT littermate mice. At least 6 ventral horns per animal were imaged and analyzed for fluorescence intensity using Adobe Photoshop CC software for both Iba-1 and GFAP staining. For both Iba-1 and GFAP quantification, the ventral horn image of the untreated hSOD1^G93A^ littermate control mouse with the most background was chosen, and the green channel input level was adjusted so that background staining was best eliminated. This value was recorded and used for each subsequent image, including those taken from the WT control littermate and the PCA-treated hSOD1^G93A^ littermate mice such that all images were adjusted by an equivalent amount. After the channel levels were adjusted, the ventral horn was outlined using the lasso tool and green channel values for mean pixel intensity and pixel area were recorded for each ventral horn image. Total pixel intensity of the green channel was obtained by multiplying the pixel intensity and pixel area. An average of the total pixel intensity was taken for each mouse. Quantification was performed twice by two different and blinded researchers. An average of the pixel intensities for each mouse was obtained. For analysis, the average total pixel intensity for the WT littermate control mouse for each group was set at 100% and the average total pixel intensities for the untreated hSOD1^G93A^ littermate control and PCA-treated hSOD1^G93A^ littermate mice were calculated as percentages relative to the WT littermate control mouse. Mean raw GFAP and Iba-1 fluorescence intensity in the lumbar spinal cord ventral horn in the untreated hSOD1^G93A^ littermate mice and WT littermate mice were also counted and statistically compared to verify a significant disease effect.

### 2.9. Nissl Staining of Spinal Cord Sections

Prior to staining, slides were allowed to equilibrate at room temperature for at least 30 min. Slides were then washed twice with 1X PBS to remove any residual OCT. Tissue was then incubated in 70% ethanol and then 95% ethanol for 3 min each. Slides were then incubated in 100% ethanol for 3 min and then 5 min, changing the ethanol between each incubation. Slides were then washed in deionized water 3–4 times. A Cresyl Violet Counterstain Solution (Bioenno Tech, Santa Anna, CA, USA) was applied to each slide for 3 min. De-staining was then performed by washing briefly in 70 mM acetic acid solution. Slides were rinsed in deionized water 3 times. Lastly, slides were incubated sequentially in solutions of 70%, 95%, and 100% ethanol as described above. Slides were mounted with ProLong Gold anti-fade reagent and were sealed with coverslips. Stained slides were stored in the dark at −20 °C until imaging took place. Two slides from each mouse spinal cord were stained for Nissl with 6 sections per slide.

### 2.10. Imaging and Quantification of Nissl-Stained Spinal Cord Sections at End Stage of the Untreated hSOD1^G93A^ Littermate

A single image of each ventral horn was captured on the 20× objective using bright field by blinded researchers. At least six ventral horns for each mouse were imaged. Any neuron greater than 20 μm in length along its longest axis was considered to be a viable alpha motor neuron. Using Adobe Photoshop CC, the contrast was set to the WT littermate and kept the same within the group to ensure consistency in staining. Next, the ruler tool was used to measure all motor neurons greater than 20 μm. For each mouse, the average number of alpha motor neurons was taken. Quantification was performed twice by two different and blinded researchers. Each average of the untreated hSOD1^G93A^ littermate and 100 mg/kg PCA-treated hSOD1^G93A^ littermate mouse were calculated as a percent of the average of the WT littermate control mouse (set at 100%). The mean numbers of alpha motor neurons in the lumbar spinal cord ventral horn in the untreated hSOD1^G93A^ littermate mice and WT littermate mice were also statistically compared to verify a significant disease effect.

### 2.11. Alpha-Bungarotoxin and VAChT Staining of Gastrocnemius Sections

Prior to staining, slides were allowed to equilibrate at room temperature for at least 30 min. Tissue sections on the slides were then outlined with a hydrophobic pen and washed twice with 1X PBS to remove any OCT. Tissue was then incubated at room temperature in blocking buffer, containing 5% (*w*/*v*) BSA and 0.2% triton-X 100 in 1X PBS for 90 min. For analysis of NMJ area, perimeter, and complexity, the slides were then incubated for 90 min with alpha-BTx conjugated to Alexa Fluor^®^ 594 (ThermoFisher Scientific Inc., Rockford, IL, USA) at a 1:200 dilution in blocking buffer containing Hoechst nuclear stain at a dilution of 1:500. For analysis of NMJ innervation, slides were stained with primary antibody to VAChT (C-terminal) (Sigma-Aldrich, St. Louis, MO, USA) which was prepared as a 1:500 dilution in PBS containing 0.2% triton-X 100 and 2% BSA (*w*/*v*). Tissue was incubated in primary antibody overnight at 4 °C. FITC-conjugated donkey anti-rabbit antibody (Jackson Immunoresearch Laboratories, West Grove, PA, USA) was then prepared at a 1:500 dilution (*v*/*v*) and alpha-BTx conjugated to Alexa Fluor^®^ 594 (ThermoFisher Scientific Inc., Rockford, IL, USA) was prepared at a 1:200 dilution in 1X PBS containing 0.2% triton-X100 and 2% BSA (*w*/*v*) and Hoechst nuclear stain (Sigma-Aldrich, St. Louis, MO, USA) at a 1:500 dilution to detect VAChT on the presynaptic axon terminal, NMJs, and to label nuclei, respectively. Slides were washed with 1X PBS 3–4 times and then mounted using ProLong Gold anti-fade reagent and sealed with coverslips. Stained slides were stored in the dark at −20 °C until imaging took place.

### 2.12. Imaging and Area, Perimeter, and Sholl Analysis Quantification of Gastrocnemius Sections Stained with Alpha-Bungarotoxin at End Stage of the Untreated hSOD1^G93A^ Littermate Mouse

Neuromuscular junctions (20–25) were imaged for each mouse by blinded researchers on the 40^X^ objective for all mice that were euthanized at end stage of the untreated hSOD1^G93A^ littermate control mice. Using the magic wand tool in Adobe Photoshop CC, the area was measured, in pixels, for each NMJ. This was achieved by outlining the outside of each NMJ, pressing “Record Measurements”, and taking the provided “Area” value. The perimeter, in pixels, of each NMJ was also measured by tracing the NMJ inside and outside, pressing “Record Measurements”, and taking the provided “Perimeter” value. An average perimeter and area were calculated for each mouse from all the values recorded for the mouse. Quantification was performed twice by two different and blinded researchers. The untreated hSOD1^G93A^ and 100 mg/kg PCA-treated hSOD1^G93A^ littermate mice averages were calculated as a percent of the WT littermate mouse, which was set at 100%. Mean NMJ pixel area and perimeter in the untreated hSOD1^G93A^ littermate mice and WT littermate mice were also statistically compared to verify a significant disease effect. The Sholl analysis was also performed on the same NMJ images that were used to analyze area and perimeter. Briefly, using ImageJ, the red (BTx) channel was separated from all channels and the NMJ was isolated using the plugins “Despeckle” and “Find Edges”. The edges of the NMJ were traced with “Simple Neurite Tracer” and “Analyze Skeleton” plugins. Each NMJ was circled by hand and pasted into a new image. The plugin “Skeletonize” was run to draw a line around the border of the NMJ. The “Sholl Analysis” plugin was run to decide a point in the center of the NMJ and to draw concentric circles around that point at a radius increasing by 10 pixels. Mean Sholl analysis values of NMJ complexity, or the number of intersections formed between the concentric circles and the skeleton of the NMJ, were reported for each untreated hSOD1^G93A^ and 100 mg/kg PCA-treated hSOD1^G93A^ littermate mouse to be taken as a percent of the WT littermate control mouse (set at 100%). Mean Sholl analysis values from untreated hSOD1^G93A^ littermate mice and WT littermate mice were also statistically compared to verify a significant disease effect.

### 2.13. Analysis of Gastrocnemius Muscle Wet Weight at 105 Days of Age

Gastrocnemius muscle from all 105-day-old mice were placed into a tared weigh boat on a standard analytical balance to obtain muscle wet weight. Weights of gastrocnemius muscle were taken from WT, 100 mg/kg PCA-treated hSOD1^G93A^, and untreated hSOD1^G93A^ littermate mice. Each gastrocnemius muscle wet weight from the untreated hSOD1^G93A^ littermate mouse and 100 mg/kg PCA-treated hSOD1^G93A^ littermate mouse was calculated as a percent of the average of the WT littermate control mouse (set at 100%).

### 2.14. Imaging of Gastrocnemius Sections Stained with Alpha-BTx and VACht at 105 Days of Age

Neuromuscular junctions (20–25) were captured on the Rhodamine channel for each mouse by blinded researchers on the 20× objective for all mice that were euthanized at 105 days of age. VACht was captured on the Alexa Fluor 488 channel for each NMJ. Representative images are shown from a WT, untreated hSOD1^G93A^ mouse, and a 100 mg/kg PCA-treated hSOD1^G93A^ mouse.

### 2.15. Imaging of Quantification of 4-HNE-Stained Spinal Cord Sections at 105 Days of Age

A single image of each ventral horn on the 20× objective was captured for each mouse. Imaging was performed by blinded researchers. Images were captured on the Alexa Fluor 488 channel (pseudo-colored red) and the exposure time was set appropriately for the untreated hSOD1^G93A^ littermate and kept constant when imaging the PCA-treated hSOD1^G93A^ and WT littermate mice. Two littermate groups were analyzed and 6 ventral horns per animal were imaged and analyzed for fluorescence intensity using Adobe Photoshop CC software. The ventral horn image of the untreated hSOD1^G93A^ littermate control mouse with the most background was chosen, and the red channel input level was adjusted so that background staining was best eliminated. This value was recorded and used for each subsequent image, including those taken from the WT control littermate and the PCA-treated hSOD1^G93A^ littermate mice such that all images were adjusted by an equivalent amount. After the channel levels were adjusted, the ventral horn was outlined using the lasso tool and red channel values for mean pixel intensity and pixel area were recorded for each ventral horn image. Total pixel intensity of the red channel was obtained by multiplying the pixel intensity and pixel area. An average of the total pixel intensity was taken for each mouse. Quantification was performed once by a blinded researcher. An average of the pixel intensities for each mouse was obtained. For analysis, the average total pixel intensity for the WT littermate control mouse for each group was set at 100% and each individual total pixel intensity for each ventral horn for the untreated hSOD1^G93A^ littermate control and PCA-treated hSOD1^G93A^ littermate mice were calculated as percentages relative to the average total pixel intensity of the WT littermate control mouse. Mean raw 4-HNE fluorescence intensity in the lumbar spinal cord ventral horn in the untreated hSOD1^G93A^ littermate mice and WT littermate mice were also counted and statistically compared to verify a significant disease effect.

### 2.16. Statistical Analysis

Histological analyses were performed on at least 6 mice per treatment group. Differences between untreated and PCA-treated hSOD1^G93A^ littermate mice for Iba-1, GFAP, and 4-HNE intensity, NMJ area and perimeter, Nissl-stained counts, and gastrocnemius muscle wet weight were analyzed using a paired *t*-test. PaGE and rotarod were analyzed using an unpaired *t*-test at each time point. Correlation analysis of PaGE data and survival were analyzed using a Pearson correlation. Mean NMJ Sholl analysis values were analyzed using a one-way analysis of variance (ANOVA) with post-hoc Tukey’s test. Survival Kaplan–Meier curves and body weight data were analyzed using a log-rank test. For all analyses, differences were statistically significant when *p* < 0.05.

## 3. Results

### 3.1. PCA Orally Administered Beginning at Disease Onset Results in a Significant Extension of Survival but Does Not Preserve Body Weight in the hSOD1^G93A^ Mouse Model of ALS

**Figure 1:**
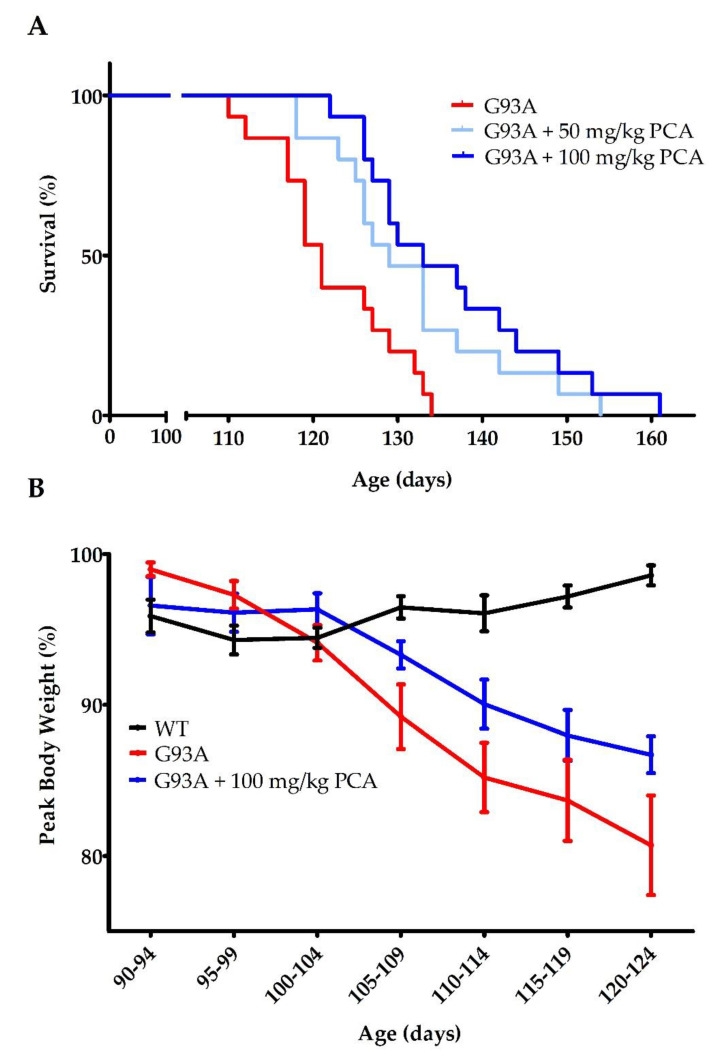
PCA treatment extends survival in the hSOD1^G93A^ mouse model of ALS. (**A**) Survival of hSOD1^G93A^ mice (untreated or treated with 50 or 100 mg/kg PCA) and wildtype mice (WT). Oral administration of either 50 or 100 mg/kg PCA beginning at disease onset (90 days of age) significantly extended median survival in hSOD1^G93A^ mice to 129 and 133 days, respectively, when compared to untreated hSOD1^G93A^ mice, which exhibited a median survival of 121 days. Curves are significantly different as determined by log-rank (Mantel–Cox) test (*p* = 0.0025; *n* = 15 mice per group). (**B**) Body weight of hSOD1^G93A^ mice (untreated or treated with 100 mg/kg PCA) and WT mice. Body weight was assessed twice per week and is expressed as the percent of peak body weight at each time point. Data are displayed as the mean ± SEM with *n* = 15 mice for each group.

In order to determine the therapeutic benefit of PCA in an ALS mouse model, we first evaluated the ability of PCA to extend the lifespan of hSOD1^G93A^ mice. Mice were dosed by oral gavage with either 50 or 100 mg/kg PCA beginning at 90 days of age. This time point corresponds with average disease onset. At this age, mice typically display gait disturbances, decreased weight, and lower limb tremors [35]. Mice were dosed by oral gavage with PCA until end stage, assessed by the ability of the mouse to right itself to sternum when placed on its side. Administration of both 50 and 100 mg/kg PCA significantly extended median survival in hSOD1^G93A^ mice to 129 and 133 days, respectively, when compared to untreated hSOD1^G93A^ mice, which exhibited a median survival of 121 days (*p* = 0.0025; Figure 1A). This impressive extension of survival indicates that PCA is slowing the disease progression in hSOD1^G93A^ ALS mice. Body weight of the hSOD1^G93A^ mice was assessed twice per week and is expressed as a percentage of peak body weight at each time point. Despite significantly extended survival, administration of PCA had no significant effect on the decline in body weight in the hSOD1^G93A^ mouse model of ALS (Figure 1B). 

### 3.2. PCA Treatment Improves Grip Strength and Motor Performance in the hSOD1^G93A^ Mouse Model of ALS

**Figure 2:**
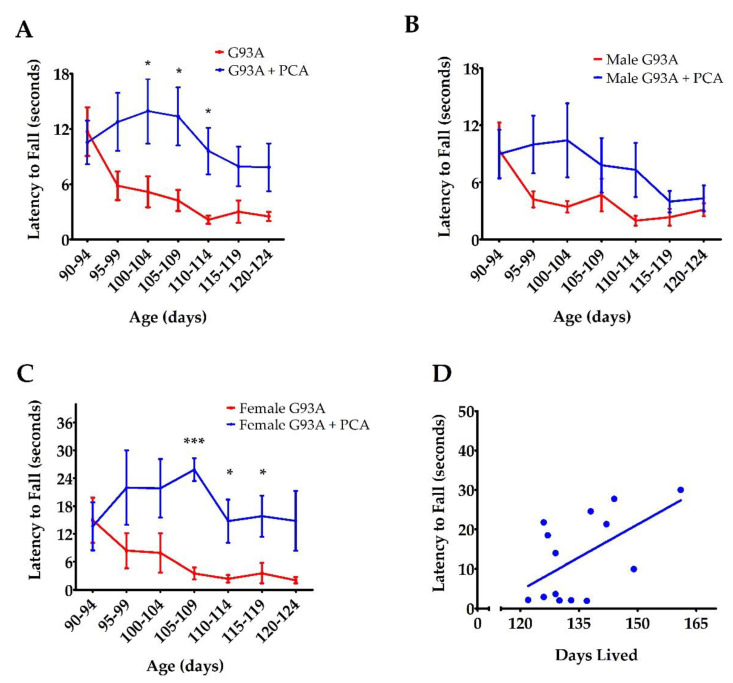
PCA treatment improves grip strength as assessed by PaGE in the hSOD1^G93A^ mouse model of ALS. (**A**) PaGE testing of hSOD1^G93A^ mice (untreated or treated with 100 mg/kg PCA) beginning at disease onset. PaGE testing was performed twice a week beginning at 90 days of age and is expressed as latency to fall. PaGE data are expressed as the mean ± SEM for each time point; *n* = 15 mice per group. * indicates *p* < 0.05 in comparison to untreated hSOD1^G93A^ littermate controls. All data were analyzed using an unpaired *t*-test at each time point. (**B**) PaGE testing of male hSOD1^G93A^ mice (untreated or treated with 100 mg/kg PCA) beginning at disease onset. PaGE data are expressed as the mean ± SEM for each time point; *n* = 9 mice per group. All data were analyzed using an unpaired *t*-test at each time point. (**C**) PaGE testing of female hSOD1^G93A^ mice (untreated or treated with 100 mg/kg PCA) beginning at disease onset. PaGE data are expressed as the mean ± SEM for each time point; *n* = 6 mice per group. All data were analyzed using an unpaired *t*-test at each time point. * indicates *p* < 0.05 in comparison to untreated hSOD1^G93A^ littermate controls. *** indicates *p* < 0.001 in comparison to untreated hSOD1^G93A^ littermate controls. (**D**) Increased average PaGE latency to fall between 100 and 114 days of age is significantly correlated with longer survival of 100 mg/kg PCA-treated hSOD1^G93A^ mice. PaGE data from 100–114 days of age are expressed as the mean for each mouse; *n* = 14 mice. All data were analyzed using a Pearson correlation (*r* = 0.558, *p* < 0.05).

Since the dose of 100 mg/kg PCA had a more pronounced effect on survival than the 50 mg/kg PCA dose, grip strength and motor function were assessed in mice dosed with 100 mg/kg PCA. hSOD1^G93A^ mice treated with 100 mg/kg PCA were further evaluated using PaGE testing and rotarod testing in order to assess motor function [36]. PaGE testing was performed twice a week beginning at disease onset until end stage of disease to assess grip strength. Administration of 100 mg/kg PCA beginning at disease onset significantly increased the latency to fall as assessed by PaGE testing at 100 to 114 days of age when compared to the untreated hSOD1^G93A^ littermate controls (*p* < 0.05, Figure 2A). PaGE data were also analyzed for differences between male and female mice. PCA-treated male hSOD1^G93A^ mice did not show a significantly increased latency to fall at any time points when compared to the untreated male hSOD1^G93A^ littermate controls (Figure 2B). However, PCA-treated male hSOD1^G93A^ mice did trend towards a significant increase in latency to fall at 95–99 (*p* = 0.118, Figure 2B) and 100–104 days of age (*p* = 0.115). PCA-treated female hSOD1^G93A^ mice displayed a significantly increased latency to fall as assessed by PaGE testing at 105–109 (*p* < 0.001, Figure 2C) and 110–119 (*p* < 0.05, Figure 2C) days of age when compared to untreated female hSOD1^G93A^ littermate controls. Lastly, we sought to understand the relationship between PaGE testing and survival in PCA-treated hSOD1^G93A^ mice. We averaged latency to fall as assessed by PaGE testing at 100–114 days of age from male and female hSOD1^G93A^ mice treated with 100 mg/kg PCA beginning at disease onset. The latency to fall at these time points was chosen to be averaged because PCA-treated hSOD1^G93A^ displayed an increased latency to fall as assessed by PaGE testing at these time points when compared to untreated hSOD1^G93A^ littermate mice. The average latency to fall over these time points was correlated with the days lived for each mouse. Average latency to fall at 100–114 days and days lived was positively and significantly correlated in PCA-treated hSOD1^G93A^ mice (r = 0.558, *p* < 0.05, Figure 2D).

**Figure 3:**
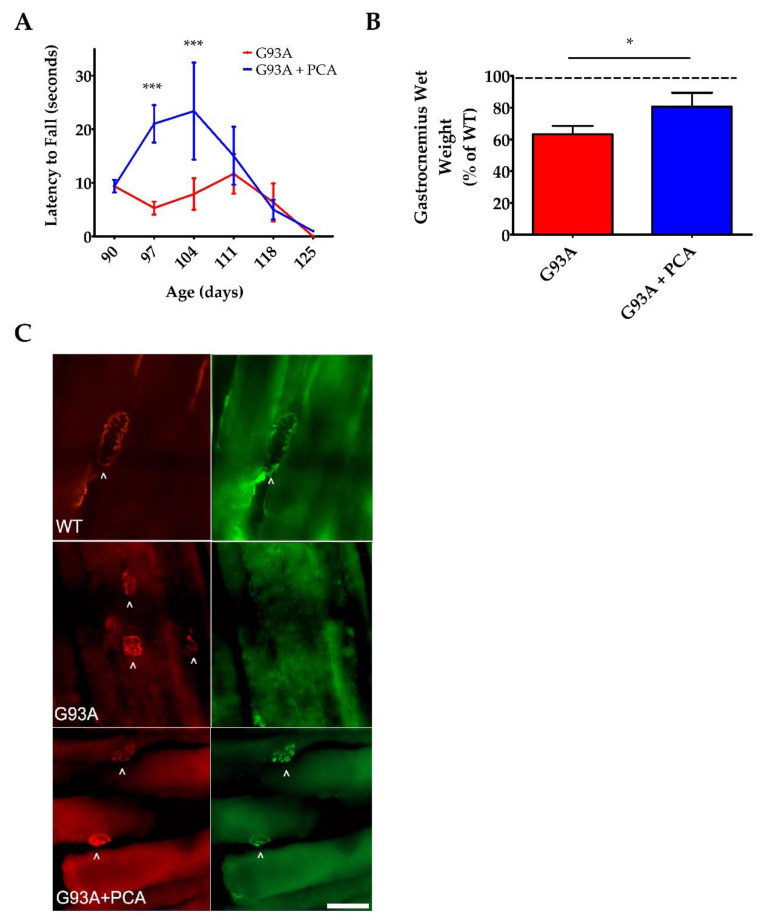
PCA treatment improves motor function as assessed by rotarod and also preserves gastrocnemius muscle weight and neuromuscular junction (NMJ) innervation at 105 days of age in the hSOD1^G93A^ mouse model of ALS. (**A**) Rotarod testing of hSOD1^G93A^ mice (untreated or treated with 100 mg/kg PCA beginning at disease onset). Rotarod testing was performed beginning at 90 days of age and extending through end stage and is expressed as latency to fall. Rotarod data are represented as the mean ± SEM for each time point; *n* = 10 mice per group. *** indicates *p* < 0.001 in comparison to untreated hSOD1^G93A^ littermate controls. All data were analyzed using an unpaired *t*-test at each time point. (**B**) Quantification of gastrocnemius muscle weights. Data are expressed as a percent of the wildtype (WT) littermate mouse muscle weight and are shown as the mean ± SEM; *n* = 7 mice per group. * indicates *p* < 0.05 compared to untreated hSOD1^G93A^ control mice (paired *t*-test). Mean gastrocnemius wet weight for the untreated hSOD1^G93A^ mouse (0.0912 ± 0.0070) is significantly decreased when compared to the WT littermate control mouse (0.1433 ± 0.0055) (*p* = 0.0002; *n* = 7 mice per group). (**C**) Representative images of gastrocnemius muscle from wildtype control mice (WT), untreated hSOD1^G93A^ mice (G93A), and hSOD1^G93A^ mice treated orally with 100 mg/kg PCA beginning at disease onset (G93A+PCA). Mice were euthanized at 105 days of age and gastrocnemius muscles were stained with alpha-BTx (red) and VAChT (green) to label NMJs and innervation of NMJs, respectively. Scale bar = 20 μm. Arrowheads point to NMJs stained positively with alpha-BTx and VAChT.

For further motor function analysis, rotarod testing was performed once a week beginning at disease onset until end stage of disease. This behavioral assay also showed an impressive enhancement in the motor function of PCA-treated hSOD1^G93A^ mice when compared to untreated hSOD1^G93A^ littermates. Administration of 100 mg/kg PCA beginning at disease onset significantly but transiently increased the latency to fall as measured by rotarod testing at 97 and 104 days of age when compared to the untreated hSOD1^G93A^ littermate control mice (*p* < 0.001, Figure 3A). The results of these behavioral tests indicate that oral administration of PCA beginning at disease onset significantly improves balance, grip strength, and motor coordination in the hSOD1^G93A^ mouse model of ALS.

### 3.3. PCA Treatment Preserves Gastrocnemius Muscle Wet Weight, Protects NMJ Innervation, and Reduces Oxidative Stress in the hSOD1^G93A^ Mouse Model of ALS at 105 Days of Age

We observed the greatest improvements in motor function as assessed by rotarod and PaGE testing between 97 and 114 days of age in PCA-treated hSOD1^G93A^ mice. Therefore, we next analyzed gastrocnemius muscle weight at 105 days of age, the time point at which we saw the greatest behavioral therapeutic effect of PCA. Wet muscle weight isolated from untreated hSOD1^G93A^ mice (0.091 g ± 0.007) was significantly decreased when compared to WT littermate mice (0.143 g ± 0.006) (*p* < 0.01, Figure 3B). PCA-treated mice exhibited a significant preservation of gastrocnemius muscle wet weight when compared to untreated hSOD1^G93A^ littermate mice. PCA-treated hSOD1^G93A^ mice had a mean muscle wet weight of 80% of the WT littermate mice, while untreated hSOD1^G93A^ mice had a mean muscle weight of only 63% of WT littermate mice (*p* < 0.05, Figure 3B). These results show that PCA treatment significantly delayed atrophy of the gastrocnemius muscle in the hSOD1^G93A^ mouse model of ALS. To support these findings, we stained gastrocnemius muscle isolated at 105 days of age with alpha-BTx and VACht in order to visualize innervated NMJs. Although these findings are preliminary, they indicate that PCA-treated mice exhibit a protection of NMJ innervation when compared to untreated hSOD1^G93A^ littermate mice, as evidenced by retention of the overlapping staining of alpha-BTx and VACht in the treated mouse NMJs (Figure 3C).

**Figure 4:**
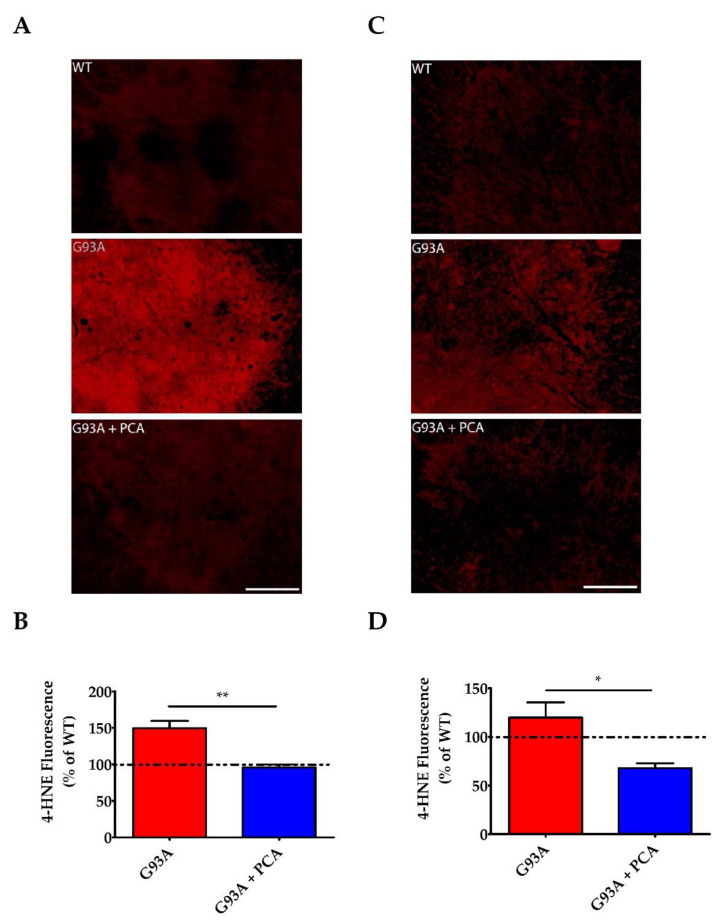
PCA treatment significantly reduces lipid peroxidation in the ventral horn of the spinal cord in the hSOD1^G93A^ mouse model of ALS. (**A**,**C**) Representative images of lumbar spinal cord ventral horns stained for 4-HNE from two littermate groups of wildtype control mice (WT), untreated hSOD1^G93A^ mice (G93A), and hSOD1^G93A^ mice treated orally with 100 mg/kg PCA beginning at disease onset (G93A+PCA). Mice were euthanized at 105 days of age and ventral horns were stained with an antibody to 4-HNE to measure lipid peroxidation. Scale bar = 70 μm. (**B**) Quantification of spinal cord ventral horns stained with 4-HNE as described and shown in A. The 4-HNE fluorescence intensity of untreated and PCA-treated hSOD1^G93A^ littermate mice were normalized and expressed as a percentage of mean 4-HNE fluorescence measured in the WT littermate control mouse. Data are expressed as the mean ± SEM; 6 ventral horns were imaged per mouse. ** indicates *p* < 0.01 compared to the untreated hSOD1^G93A^ control littermate (paired *t*-test). Raw mean 4-HNE fluorescence units for the untreated hSOD1^G93A^ mouse (11.70 ± 0.78) × 10^6^) are significantly higher than the WT littermate mouse (7.83 ± 0.16) × 10^6^) (*p* < 0.01) (**D**) Quantification of spinal cord ventral horns stained with 4-HNE as described and shown in C. The 4-HNE fluorescence intensity of untreated and PCA-treated hSOD1^G93A^ littermate mice were normalized and expressed as a percentage of mean 4-HNE fluorescence measured in the WT littermate control mouse. Data are expressed as the mean ± SEM; 6 ventral horns were imaged per mouse. * indicates *p* < 0.05 compared to the untreated hSOD1^G93A^ control littermate (paired *t*-test). Raw mean 4-HNE fluorescence units for the untreated hSOD1^G93A^ mouse (6.56 ± 0.88) × 10^6^) are significantly higher than the WT (4.17 ± 0.24) × 10^6^) (*p* < 0.05).

Oxidative stress is an underlying pathology of ALS and contributes to motor neuron death and subsequent skeletal muscle atrophy and deficits in motor function [16,17]. Therefore, we also analyzed the effect of PCA on the production of 4-HNE in the lumbar spinal cord ventral horn of the hSOD1^G93A^ mouse model of ALS. Lumbar spinal cord isolated at 105 days of age from two littermate groups was stained with antibodies against 4-HNE to measure lipid peroxidation. Untreated hSOD1^G93A^ mice in both groups exhibit significantly higher raw 4-HNE fluorescence units (((11.70 ± 0.78) × 10^6^) and ((6.56 ± 0.88) × 10^6^))) when compared to their WT littermate control mice (((7.83 ± 0.16) × 10^6^) and ((4.17 ± 0.24) × 10^6^))), respectively. These data indicate profound lipid peroxidation in the ventral horn (*p* < 0.01 and *p* < 0.05, Figure 4A and C, respectively). Administration of 100 mg/kg PCA beginning at disease onset significantly reduced lipid peroxidation in the ventral horn of the lumbar spinal cord in the hSOD1^G93A^ mouse model of ALS relative to the untreated littermate control (*p* < 0.01 and *p* < 0.05, Figure 4B and D, respectively).

### 3.4. PCA Treatment Significantly Preserves Motor Neurons in the Ventral Horn of the Spinal Cord in the hSOD1^G93A^ Mouse Model of ALS

**Figure 5:**
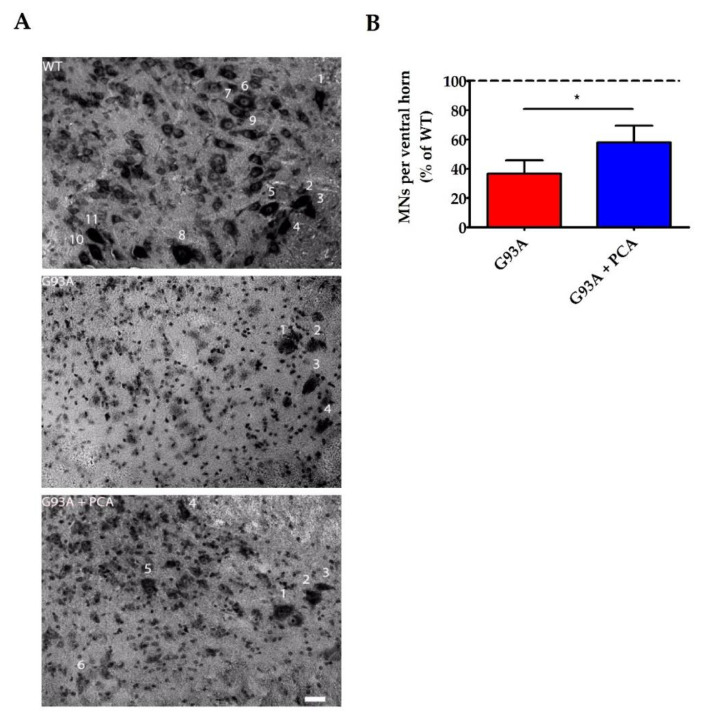
PCA treatment significantly preserves motor neurons in the ventral horn of the spinal cord in the hSOD1^G93A^ mouse model of ALS. (**A**) Representative images of lumbar spinal cord ventral horns from wildtype control mice (WT), untreated hSOD1^G93A^ control mice (G93A) and hSOD1^G93A^ mice treated orally with 100 mg/kg PCA beginning at disease onset (G93A+PCA). Mice were euthanized at end stage of the untreated hSOD1^G93A^ littermate control mouse and ventral horns were Nissl stained to label neuronal cell bodies. Stained soma were measured along the longest axis and cells were considered alpha motor neurons if the length was greater than 20 μm. Scale bar = 20 μm. (**B**) Quantification of Nissl-stained alpha motor neurons as described in A. The number of alpha motor neurons in the ventral horns of lumbar spinal cord of untreated and PCA-treated hSOD1^G93A^ littermate mice were normalized and expressed as a percentage of the number of alpha motor neurons measured in the WT littermate control mouse. Data are expressed as the mean ± SEM; *n* = 7 mice per group; 4–6 ventral horns were imaged per mouse. * indicates *p* < 0.05 compared to untreated hSOD1^G93A^ littermate controls (paired *t*-test). Mean number of alpha motor neurons present in the ventral horn lumbar spinal cord for the untreated hSOD1^G93A^ littermate mouse (1.92 ± 0.47) is significantly less than that of the WT (5.55 ± 0.57) (*p* = 0.004; *n* = 7 mice per group). Abbreviations used: MNs = motor neurons.

It was evident that PCA had a beneficial therapeutic effect, as evidenced by the extension of survival and motor function improvements in the hSOD1^G93A^ mouse model of ALS. However, the effects of PCA on inflammation, motor neuron preservation, and neuromuscular junction integrity needed to be studied in order to support the survival and behavioral assay results. Lumbar spinal cord was isolated at end stage of the untreated hSOD1^G93A^ littermate control mouse and Nissl staining was performed so that neuronal cell bodies could be identified. The number of alpha motor neurons, with somas typically greater than 20 μm along the longest axis, were counted in the ventral horn of the lumbar spinal cord of untreated and PCA-treated hSOD1^G93A^ littermate mice. These values were normalized and expressed as a percentage of the number of alpha motor neurons counted in the WT littermate control mouse. The cell bodies of alpha motor neurons are located in the ventral horn of the lumbar spinal cord and their axons project to innervate skeletal muscle fibers of the leg muscles including the gastrocnemius muscle. The hSOD1^G93A^ mouse model of ALS exhibits a rapidly progressive lower limb muscular atrophy and subsequent paralysis as a result of alpha motor neuron death in the ventral horn of the spinal cord and retraction of motor axons away from the NMJs [35]. As anticipated, untreated hSOD1^G93A^ mice exhibited nearly 60% fewer alpha motor neurons in the lumbar spinal cord ventral horn than their healthy WT littermates (Figure 5A). However, when 100 mg/kg PCA was administered beginning at disease onset, the average alpha motor neuron count in the ventral horn of the lumbar spinal cord was significantly increased in comparison to the untreated hSOD1^G93A^ littermate mouse (*p* < 0.05, Figure 5B). These findings may contribute to the observed improvements in motor function as assessed by rotarod and PaGE testing.

### 3.5. PCA Treatment Significantly Reduces Astrogliosis and Microgliosis in the Ventral Horn of the Spinal Cord in the hSOD1^G93A^ Mouse Model of ALS

**Figure 6:**
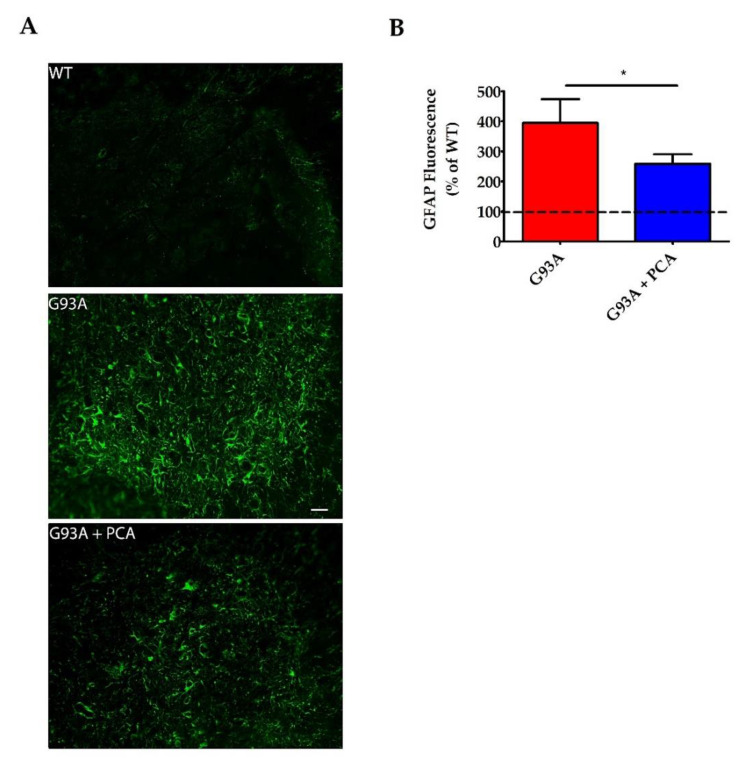
PCA treatment significantly reduces astrogliosis in the ventral horn of the spinal cord in the hSOD1^G93A^ mouse model of ALS. (**A**) Representative images of lumbar spinal cord ventral horns stained for GFAP from wildtype control mice (WT), untreated hSOD1^G93A^ mice (G93A), and hSOD1^G93A^ mice treated orally with 100 mg/kg PCA beginning at disease onset (G93A+PCA). Mice were euthanized at end stage of the untreated hSOD1^G93A^ littermate control mouse and ventral horns were stained with an antibody to GFAP to label astrocytes. Scale bar = 20 μm. (**B**) Quantification of spinal cord ventral horns stained with GFAP as described in A. GFAP fluorescence intensity of untreated and PCA-treated hSOD1^G93A^ littermate mice were normalized and expressed as a percentage of GFAP fluorescence measured in the WT littermate control mouse. Data are expressed as the mean ± SEM; n = 10 mice per group; 4–6 ventral horns were imaged per mouse. * indicates *p* < 0.05 compared to untreated hSOD1^G93A^ controls (paired *t*-test). Raw mean GFAP fluorescence units for the untreated hSOD1^G93A^ mice ((3.21 ± 0.46) × 10^6^) are significantly higher than the WT ((0.97 ± 0.16) × 10^6^) (*p* = 0.001; *n* = 10 mice per group).

**Figure 7:**
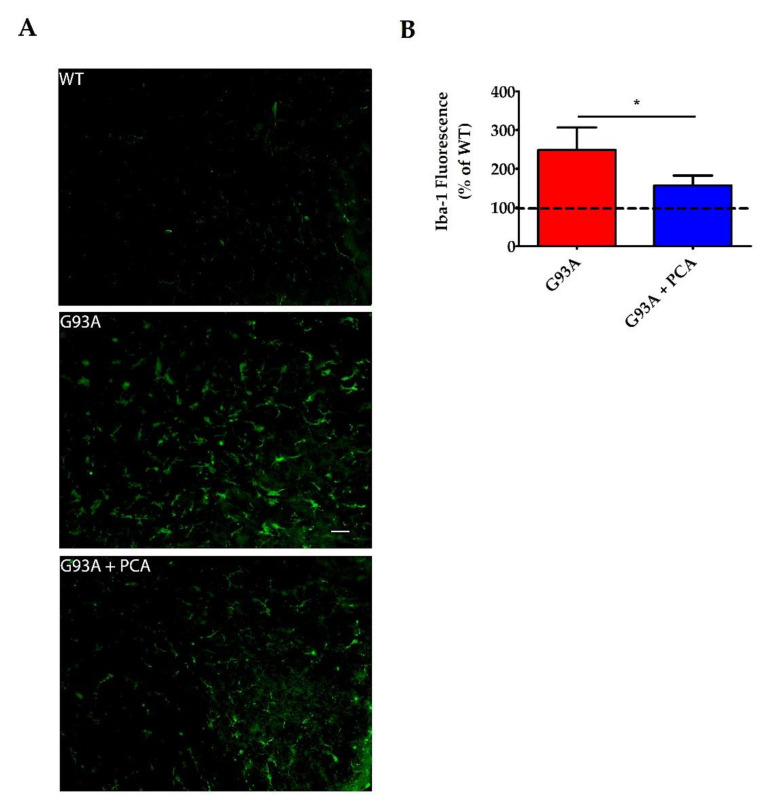
PCA treatment significantly reduces microgliosis in the ventral horn of the spinal cord in the hSOD1^G93A^ mouse model of ALS. (**A**) Representative images of lumbar spinal cord ventral horns stained for Iba-1 from wildtype control mice (WT), untreated hSOD1^G93A^ mice (G93A), and hSOD1^G93A^ mice treated orally with 100 mg/kg PCA beginning at disease onset (G93A+PCA). Mice were euthanized at end stage of the untreated hSOD1^G93A^ littermate mouse and ventral horns were stained with an antibody to Iba-1 to label microglia. Scale bar = 20 μm. (**B**) Quantification of spinal cord ventral horns stained with Iba-1 as described in A. Iba-1 fluorescence intensity of untreated and PCA-treated hSOD1^G93A^ littermate mice were normalized and expressed as a percentage of Iba-1 fluorescence measured in the WT littermate control mouse. Data are expressed as the mean ± SEM; *n* = 6 mice per group; 4–6 ventral horns were imaged per mouse. * indicates *p* = 0.05 compared to untreated hSOD1^G93A^ controls (paired *t*-test). Raw mean Iba-1 fluorescence units for the untreated hSOD1^G93A^ mouse ((3.41 ± 0.52) × 10^6^) are significantly higher than the WT ((1.56 ± 0.29) × 10^6^) (*p* < 0.05; *n* = 6 mice per group).

A robust neuroinflammatory response in the central nervous system of the hSOD1^G93A^ mouse model contributes to the motor neuron death and muscle atrophy producing the ALS-like phenotype [35]. Therefore, we next analyzed the effect of PCA on astrogliosis and microgliosis in the lumbar spinal cord ventral horn of the hSOD1^G93A^ mouse model of ALS. Lumbar spinal cord isolated at end stage of the untreated hSOD1^G93A^ littermate mouse was stained with antibodies against GFAP and Iba-1 to identify astrocytes and microglia, respectively. Untreated hSOD1^G93A^ mice exhibit significantly higher raw mean GFAP fluorescence units ((3.21 ± 0.46) × 10^6^) when compared to WT littermate control mice ((0.97 ± 0.16) × 10^6^) indicating profound astrogliosis in the ventral horn (*p* = 0.001, Figure 6A). Microgliosis is also present in the lumbar spinal cord ventral horn of untreated hSOD1^G93A^ mice, as these mice exhibited significantly higher raw mean Iba-1 fluorescence units ((3.41 ± 0.52) × 10^6^) when compared to healthy WT littermate control mice ((1.56 ± 0.29) × 10^6^) (*p* < 0.05, Figure 7A). Administration of 100 mg/kg PCA beginning at disease onset significantly reduced astrogliosis in the ventral horn of the lumbar spinal cord in the hSOD1^G93A^ mouse model of ALS from a mean 394% increase to a mean 258% increase in GFAP fluorescence units relative to the WT littermate control (*p* < 0.05, Figure 6B). Administration of PCA also significantly reduced microgliosis in the ventral horn lumbar spinal cord in the hSOD1^G93A^ mouse model of ALS from a mean 248% increase to a mean 156% increase in Iba-1 fluorescence units relative to the WT littermate control (*p* < 0.05, Figure 7B). From these data, we conclude that PCA significantly reduces gliosis which may ultimately protect alpha motor neurons in the ventral horn of the lumbar spinal cord in the hSOD1^G93A^ mouse model of ALS.

### 3.6. PCA Treatment Significantly Preserves Neuromuscular Junctions of the Gastrocnemius Muscle in the hSOD1^G93A^ Mouse Model of ALS

**Figure 8:**
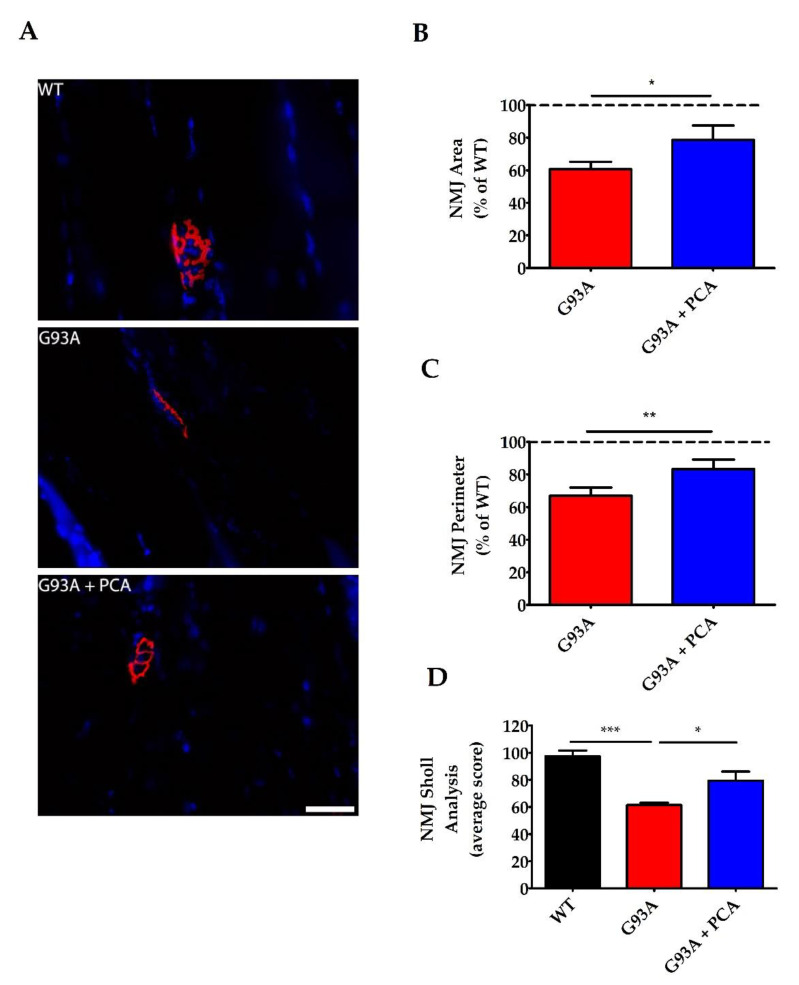
PCA treatment significantly preserves neuromuscular junctions (NMJ) in the gastrocnemius muscle in the hSOD1^G93A^ mouse model of ALS. (**A**) Representative images of gastrocnemius muscle from wildtype control mice (WT), untreated hSOD1^G93A^ mice (G93A), and hSOD1^G93A^ mice treated orally with 100 mg/kg PCA beginning at disease onset (G93A+PCA). Mice were euthanized at end stage of the untreated hSOD1^G93A^ littermate mouse and gastrocnemius muscles were stained with alpha-BTx (red) and Hoechst (blue) to label NMJs and nuclei, respectively. Scale bar = 40 μm. (**B**) Quantification of gastrocnemius NMJ area stained with alpha bungarotoxin as described in A. The NMJ area, measured in pixels by tracing the outside of the neuromuscular junction, of untreated and PCA-treated hSOD1^G93A^ littermate mice were normalized and expressed as a percentage of NMJ area measured in the WT littermate control mouse. Data are expressed as the mean ± SEM; *n* = 8 mice per group; 20–25 NMJs were imaged per mouse. * indicates *p* < 0.05 compared to untreated hSOD1^G93A^ control mice (paired *t*-test). Mean NMJ pixel area for the untreated hSOD1^G93A^ littermate mouse (11,842 ± 1204) is significantly less than the WT littermate control mouse (17,966 ± 818) (*p* = 0.01; *n* =8 mice per group). (**C**) Quantification of gastrocnemius NMJ perimeter stained with alpha bungarotoxin as described in A. The NMJ perimeter, measured in pixels by tracing the inside and outside of the NMJ, of untreated and PCA-treated hSOD1^G93A^ littermate mice were normalized and expressed as a percentage of NMJ perimeter measured in the WT littermate control mouse. Data are expressed as the mean ± SEM; *n* = 9 mice per group; 20–25 neuromuscular junctions were imaged per mouse. ** indicates *p* < 0.01 compared to untreated hSOD1^G93A^ controls (paired *t*-test). Mean NMJ pixel perimeter for the untreated hSOD1^G93A^ littermate mouse (730.9 ± 39.37) is significantly less than the WT littermate control mouse (1141 ± 94.52) (*p* = 0.0007; *n* = 9 mice per group). (**D**) Quantification of gastrocnemius NMJ Sholl analysis stained with alpha bungarotoxin as described in A. WT, untreated, and PCA-treated hSOD1^G93A^ littermate mice were given a mean Sholl analysis value, which represents the number of intersections that the NMJ makes with concentric circles every 10 pixels from a center point. Data are expressed as the mean ± SEM; *n* = 8 mice per group; 20–25 neuromuscular junctions were imaged per mouse. * indicates *p* < 0.05 and *** indicates *p* < 0.001 compared to WT littermate control mice (one-way ANOVA with post-hoc Tukey’s test).

After determining that PCA has neuroprotective and anti-inflammatory effects in the ventral horn of the lumbar spinal cord isolated from hSOD1^G93A^ mice, we sought to analyze NMJs in gastrocnemius muscle in order to further elucidate how administration of PCA improved motor deficits. Neuromuscular junctions represent the synapse between the alpha motor neuron axon terminal and the gastrocnemius muscle fiber. In the hSOD1^G93A^ mouse model of ALS, NMJs become weakened and break down as the alpha motor neuron cell body dies and the axon retracts away from the muscle. This results in skeletal muscle atrophy and paralysis characteristically seen in this mouse model [35]. To analyze the NMJs, gastrocnemius muscle was isolated at end stage of the untreated hSOD1^G93A^ littermate mouse and stained with alpha-bungarotoxin. Alpha-bungarotoxin binds to nicotinic acetylcholine receptors found on the gastrocnemius muscle. Mean NMJ area, perimeter, and Sholl analysis values of untreated and PCA-treated hSOD1^G93A^ littermate mice were normalized and expressed as a percentage of the corresponding NMJ values measured in the WT littermate control mouse. Untreated hSOD1^G93A^ mice exhibited an overall decreased NMJ pixel area (11,842 ± 1204) when compared to WT littermate control mice (17,966 ± 818) (*p* = 0.01, Figure 8A). Furthermore, untreated hSOD1^G93A^ mice also exhibited an overall decreased NMJ pixel perimeter (731 ± 40) when compared to the healthy WT littermate control mice (1141 ± 95) (*p* = 0.0007, Figure 8A). Administration of 100 mg/kg PCA beginning at disease onset significantly preserved NMJ area yielding an average NMJ pixel area of 79% of the WT littermate mice compared to the untreated hSOD1^G93A^ littermate mice, which had an average area of 61% of the WT littermate mice (*p* < 0.05, Figure 8B). Furthermore, PCA significantly preserved NMJ perimeter in the hSOD1^G93A^ mouse model of ALS. PCA-treated hSOD1^G93A^ mice exhibited a mean NMJ pixel perimeter of 81% of WT littermate mice versus untreated hSOD1^G93A^ littermate mice, which exhibited a mean of 64% of WT littermate mice (*p* < 0.05, Figure 8C). Untreated hSOD1^G93A^ mice also exhibited an overall decreased NMJ mean Sholl analysis value (62 ± 1.6) when compared to WT littermate mice (97 ± 4.3) (*p* < 0.001, Figure 8D). More importantly, PCA-treated hSOD1^G93A^ littermates exhibited a significantly greater mean Sholl analysis value (79 ± 6.8) when compared to untreated hSOD1^G93A^ littermate mice (*p* < 0.05, Figure 8D). These findings indicate that PCA protects the synapse between the alpha motor neuron axon and the gastrocnemius muscle, ultimately allowing for improved motor function and mobility.

## 4. Discussion

Plant-derived polyphenolic compounds exhibit anti-inflammatory, antioxidant, and neuroprotective capabilities; therefore, they have the potential to be safe, cost-effective, and successful agents in treating neurodegenerative diseases such as ALS. The catechol, protocatechuic acid (PCA), is a phenolic acid metabolite of kuromanin, an anthocyanin found in foods such as blackberries, bilberries, and black rice. Here, we demonstrate that PCA extends survival, improves motor function, reduces gliosis, protects motor neurons, and preserves NMJs in the hSOD1^G93A^ mouse model of ALS.

To ensure that PCA was non-toxic and had therapeutic potential, we first studied the effects of PCA on survival. We found that daily administration of 100 mg/kg PCA by oral gavage beginning at disease onset significantly extended survival of hSOD1^G93A^ mice when compared to untreated hSOD1^G93A^ littermate control mice. PCA-treated hSOD1^G93A^ mice also exhibited significantly improved motor function as assessed by rotarod and PaGE testing when compared to untreated hSOD1^G93A^ littermate control mice. To supplement these findings, we also analyzed gastrocnemius muscle weight at 105 days of age, a time point at which we observed significant peak motor performance in PCA-treated hSOD1^G93A^ mice. We found a preservation of muscle wet weight in PCA-treated hSOD1^G93A^ littermate mice when compared to untreated littermate controls at 105 days of age. At this time point, we also observed that PCA-treated hSOD1^G93A^ mice seemed to display a preservation of NMJ innervation in gastrocnemius muscle when compared to untreated hSOD1^G93A^ mice. Indeed, PCA administration preserved motor function and muscle weight well into the disease course, indicating that this compound was able to slow the progression of motor symptoms, which could indicate improved quality of life.

We next sought to determine how PCA was able to elicit a significant improvement in survival and motor function. We analyzed the ventral horn of the lumbar spinal cord at end stage of untreated hSOD1^G93A^ littermate mice and found that PCA-treated hSOD1^G93A^ mice had significantly reduced astrogliosis, microgliosis, and increased motor neuron count when compared to untreated hSOD1^G93A^ littermate control mice. Furthermore, we analyzed NMJs within the gastrocnemius muscle isolated from PCA-treated hSOD1^G93A^ mice at end stage of the untreated hSOD1^G93A^ littermate control mice and measured them in terms of overall area, perimeter, and complexity (via Sholl analysis). We found that oral treatment with PCA significantly preserved NMJ area, perimeter, and complexity when compared to untreated hSOD1^G93A^ littermate control mice. Furthermore, at 105 days of age, we analyzed gastrocnemius muscle stained with alpha-BTx along with antibodies against VAChT. We wanted to visualize the NMJs and their innervation by presynaptic cholinergic neurons. Representative images indicate that administration of PCA was able to protect the innervation of the NMJ at this time point. Although we did not quantitatively assess NMJ innervation, the combined results of preserved NMJ size and complexity at end stage of the untreated hSOD1^G93A^ littermate control mice, along with a preservation of gastrocnemius muscle wet weight and improved motor performance at 105 days of age, indicate that PCA has a beneficial effect on preserving skeletal muscle performance. Taken together, these findings indicate that PCA exhibits neuroprotective properties while also reducing gliosis in vivo and therefore, should be further explored as a therapeutic for ALS.

In future studies, we plan to further examine the mechanism of action of PCA in mitigating the deleterious effects of ALS. We have previously shown that PCA exhibits antioxidant activity due to its catechol structure. PCA has the ability to chelate metal ions, act as a reducing agent, and scavenge free radical species including nitric oxide [29,32]. In the current study, we performed a preliminary analysis and explored the effect of PCA on lipid peroxidation in the ventral horn of lumbar spinal cord isolated at 105 days of age from two groups of WT, untreated hSOD1^G93A^, and PCA-treated hSOD1^G93A^ littermate mice. Ventral horn was stained with 4-HNE, a byproduct of lipid peroxidation, and fluorescence intensity was measured. These data show that untreated hSOD1^G93A^ mice had significantly higher levels of 4-HNE fluorescence intensity in the ventral horn when compared to their WT littermates, a result that has previously been shown by others [37]. Furthermore, PCA-treated hSOD1^G93A^ mice exhibited significantly lower levels of 4-HNE fluorescence intensity when compared to their untreated hSOD1^G93A^ littermate controls. These data are further supported by previous research indicating that PCA has the ability to protect cells from mitochondrial dysfunction and apoptosis in vitro and in vivo [38,39]. Furthermore, PCA is able to increase glutathione and superoxide dismutase activity and decrease lipid peroxidation in vitro [40,41]. An increase in free radical species, release of pro-inflammatory cytokines by microglia and astrocytes, and mitochondrial dysfunction all contribute to the oxidative stress burden seen in ALS patients [18,23,24,42]. Consequently, markers such as glutathione peroxidase and malondialdehyde have been found to be elevated in the serum, plasma, and urine of ALS patients [20,21]. Research has demonstrated that oxidative stress heavily contributes to motor neuron death in ALS and PCA has been studied for its antioxidant properties. Therefore, it is possible that PCA is aiding in the preservation of motor neuron viability by reducing the oxidative stress burden through free radical scavenging or by an upregulation of endogenous antioxidant activity. Each of these potential mechanisms should be evaluated in future studies.

Acting in parallel with oxidative stress in the pathogenesis of ALS is neuroinflammation. For example, mutant SOD1 contributes to the death of motor neurons and promotes microgliosis and astrogliosis in the spinal cord. In the hSOD1^G93A^ mouse model of ALS, glial cells, such as astrocytes and microglia, overexpress mutant SOD1. This is toxic to motor neurons and causes an accelerated disease progression. It is theorized that ALS disease onset induced by expression of mutant SOD1 is non-cell autonomous and that glial cells play a central role in motor neuron death [43,44]. Microglial cells expressing mutant SOD1 become activated and release pro-inflammatory cytokines and free radical species [10,45]. Furthermore, mutant SOD1-expressing microglia release increased nitric oxide, superoxide, and decreased insulin-like growth factor-1 when compared to WT microglia in the presence of lipopolysaccharide [12]. Neuroinflammatory microglia also contribute to the activation of astrocytes. Activated astrocytes lose the ability to promote motor neuron survival, phagocytosis, and synaptogenesis [11]. Furthermore, activated astrocytes expressing mutant SOD1 contribute to the death of primary spinal motor neurons [46]. In vitro, PCA has the ability to reduce pro-inflammatory cytokines and nitric oxide production in lipopolysaccharide treated microglial cultures [34,47]. In vivo, PCA treatment reduces cyclooxygenase-2, interleukin-1β, interleukin-6, tumor necrosis factor-α, and prostaglandin E2 expression in inflammatory models in rats and mice [40,47,48]. In the hSOD1^G93A^ mouse model of ALS, we have found that PCA significantly reduces both astrogliosis and microgliosis in the ventral horn of the lumbar spinal cord. While further study is required to determine whether reducing the presence of these cells also reduces the release of pro-inflammatory factors, our data suggest that the reduction in the presence of reactive astrocytes and microglia could be a principal mechanism by which PCA preserves motor neuron survival and overall motor function in hSOD1^G93A^ mice.

In the hSOD1^G93A^ mouse model of ALS, NMJs become weakened and break down as the alpha motor neuron cell body dies and the axon retracts away from the muscle. This results in skeletal muscle atrophy and paralysis characteristically seen in this mouse model. Since PCA-treated hSOD1^G93A^ mice exhibited neuroprotection in the ventral horn of the lumbar spinal cord, we aimed to explore the preservation of the NMJ which represents the synapse between the alpha motor neuron axon terminal and the gastrocnemius muscle fiber. In the hSOD1^G93A^ mouse model of ALS, detachment of nerve terminals from the neuromuscular junction can be seen as early as 10 weeks of age [49]. Although no previous research has explored the effect of PCA treatment on NMJs in mice, we found that PCA was able to preserve the size and complexity of NMJs in hSOD1^G93A^ mice. These findings help further explain the improved motor function measured by rotarod and PaGE testing in PCA-treated hSOD1^G93A^ mice. However, the precise mechanism by which PCA protects the NMJs is currently unknown.

This study is valuable in that it highlights the ability of PCA to significantly reduce neuronal death and gliosis in a mouse model of ALS. However, the therapeutic benefits of PCA are not limited to ALS. Oxidative stress and neuroinflammation contribute to the pathology and subsequent neuronal death observed in many neurodegenerative diseases such as Alzheimer’s disease (AD) and Parkinson’s disease (PD). PCA has been previously studied in mouse models of AD and was able to improve spatial learning, decrease inflammatory cytokine expression, and increase expression of brain-derived neurotrophic factor in the APP/PS1 mouse model of familial AD [50]. Furthermore, a diet high in date palm fruits, which contain high amounts of phenolic compounds, including PCA, improved spatial learning deficits, and resulted in a reduction in lipid peroxidation and restoration of antioxidant enzymes in the [APPsw]/Tg2576 mouse model of AD [51,52]. In cell models of PD, PCA treatment resulted in a significant upregulation of antioxidant enzymes and inhibited the activation of nuclear factor-κB and expression of inducible nitric oxide synthase [53]. PCA was also able to prevent apoptosis, reduce reactive oxygen species (ROS) production, decrease activation of caspase-3, and enhance SOD activity in in vitro models of PD [54,55]. In vivo, PCA was able to improve motor function and ameliorate PD pathology in the substantia nigra in both MPTP and 6-hydroxydopamine mouse models of PD [53,56]. Interestingly, PCA has been shown in vitro to inhibit aggregation of pathogenic proteins including amyloid beta peptide and alpha-synuclein [57]. Therefore, it will be of interest in future studies to determine whether PCA attenuates aggregation of mutant SOD1 or TDP-43 in models of ALS.

## 5. Conclusions

This preclinical study is the first to explore the therapeutic benefits of PCA in a mouse model of ALS. Our findings of the neuroprotective and anti-inflammatory effects of PCA in the hSOD1^G93A^ mouse model of ALS are supported by previous studies showing similar effects of PCA in other models of neurodegeneration. Although PCA has been well studied in other disease models (e.g., AD and PD), it should be further studied in additional models of ALS, such as C9orf72 and TDP-43 ALS, to further elucidate its benefit for treating ALS in a diverse patient population. A thorough analysis of the effect of PCA treatment on levels of biomarkers relating to oxidative stress and neuroinflammation should also be performed. It would be important to measure the effect of PCA treatment on levels of pro-inflammatory cytokines and intracellular levels of ROS (superoxide anion, nitric oxide) in lumbar spinal cord and gastrocnemius muscle in the hSOD1^G93A^ model and other mouse models of ALS, and to analyze these levels over the time course of the disease. Such an analysis would allow for identification of predictive biomarkers for the efficacy of PCA treatment and ALS disease progression. Furthermore, other phenolic acid metabolites of anthocyanin compounds (e.g., 4-hydroxybenzoic acid, gallic acid, and syringic acid) should be studied for similar benefits in ALS and other neurodegenerative diseases. Our findings indicate that nutraceutical phenolic compounds, such as PCA, have the potential to help treat patients with ALS and should be investigated as possible therapeutics for this devastating disorder.
